# Distribution of item responses and total item scores for the Center for Epidemiologic Studies Depression Scale (CES-D): Data from the Irish Longitudinal Study on Ageing (TILDA)

**DOI:** 10.1371/journal.pone.0202607

**Published:** 2018-08-16

**Authors:** Shinichiro Tomitaka, Yohei Kawasaki, Kazuki Ide, Maiko Akutagawa, Yutaka Ono, Toshiaki A. Furukawa

**Affiliations:** 1 Department of Mental Health, Panasonic Health Center, Tokyo, Japan; 2 Department of Health Promotion and Human Behavior, Kyoto University Graduate School of Medicine/School of Public Health, Kyoto, Japan; 3 Clinical Research Center, Chiba University Hospital, Japan; 4 Department of Pharmacoepidemiology, Graduate School of Medicine and Public Health, Kyoto University, Kyoto, Japan; 5 Center for the Promotion of Interdisciplinary Education and Research, Kyoto University, Kyoto, Japan; 6 Department of Drug Evaluation and Informatics, School of Pharmaceutical Sciences, University of Shizuoka, Shizuoka, Japan; 7 Center for the Development of Cognitive Behavior Therapy Training, Tokyo, Japan; Chiba Daigaku, JAPAN

## Abstract

**Background:**

Previous studies have shown that item responses and total scores on depression screening scales follow characteristic distribution patterns in the United States and Japanese general populations. However, the degree to which these findings, especially in terms of item responses, can be generalized to a European population is unknown. Thus, we analyzed the item responses and total score distribution for the Center for Epidemiologic Studies Depression Scale (CES-D) in a representative Irish cohort from a large, recent study—the Irish Longitudinal Study on Ageing (TILDA).

**Methods:**

We used CES-D data from the 2009–2011 TILDA (8504 individuals). Responses for the 16 depressive symptoms included “rarely,” “some of the time,” “occasionally,” and “all of the time.” Item response patterns and total score distribution across these 16 depressive symptom items were examined using graphical analyses and exponential regression modeling.

**Results:**

Lines for item responses followed the same pattern across the 16 items. These lines were characterized by intersections in the vicinity of a single point between “rarely” and “some of the time” and parallel patterns from “some of the time” to “all of the time” on a log-normal scale. Total scores for the 16 items exhibited an exponential pattern, except for at the lower end of the distribution.

**Conclusions:**

The present findings suggest that item responses and total scores on depression screening scales among the general population follow the same characteristic patterns across populations from multiple nations.

## Introduction

Depression is a common but serious mental disorder and a major cause of disability worldwide [1]. Because the diagnosis of clinical depression is determined by the severity of depressive symptoms, researchers have paid considerable attention to the severity distribution of associated symptoms in the general population [2,3]. To date, numerous epidemiological studies have characterized responses to a variety of self-reported depression screening scales [4–6]. These studies have provided evidence of the prevalence of clinical depression among the general population. However, little attention has been paid to the mathematical patterns that item responses and total score distributions follow in these scales. These distributional patterns are important because they provide a framework for understanding how depressive symptoms are distributed across the general population. Moreover, the mathematical patterns that item responses and total score distributions follow often determine which statistical models can be used in further inference statistics. However, there are very few reports inductively identifying a reproducible distributional pattern using depression screening scales.

The Center for Epidemiologic Studies Depression Scale (CES-D) is a is a self-reported depression screening scale widely employed in population studies and in primary care [7]. The CES-D is comprised of 16 depressive symptom items and four positive affect items. This scale allows respondents to self-rate their degree of experience with each item over the preceding week using a four-point scale: “rarely” (less than 1 day), “some of the time” (1–2 days), “occasionally” (3–4 days), and “all of the time” (5–7 days).

**Fig 1:**
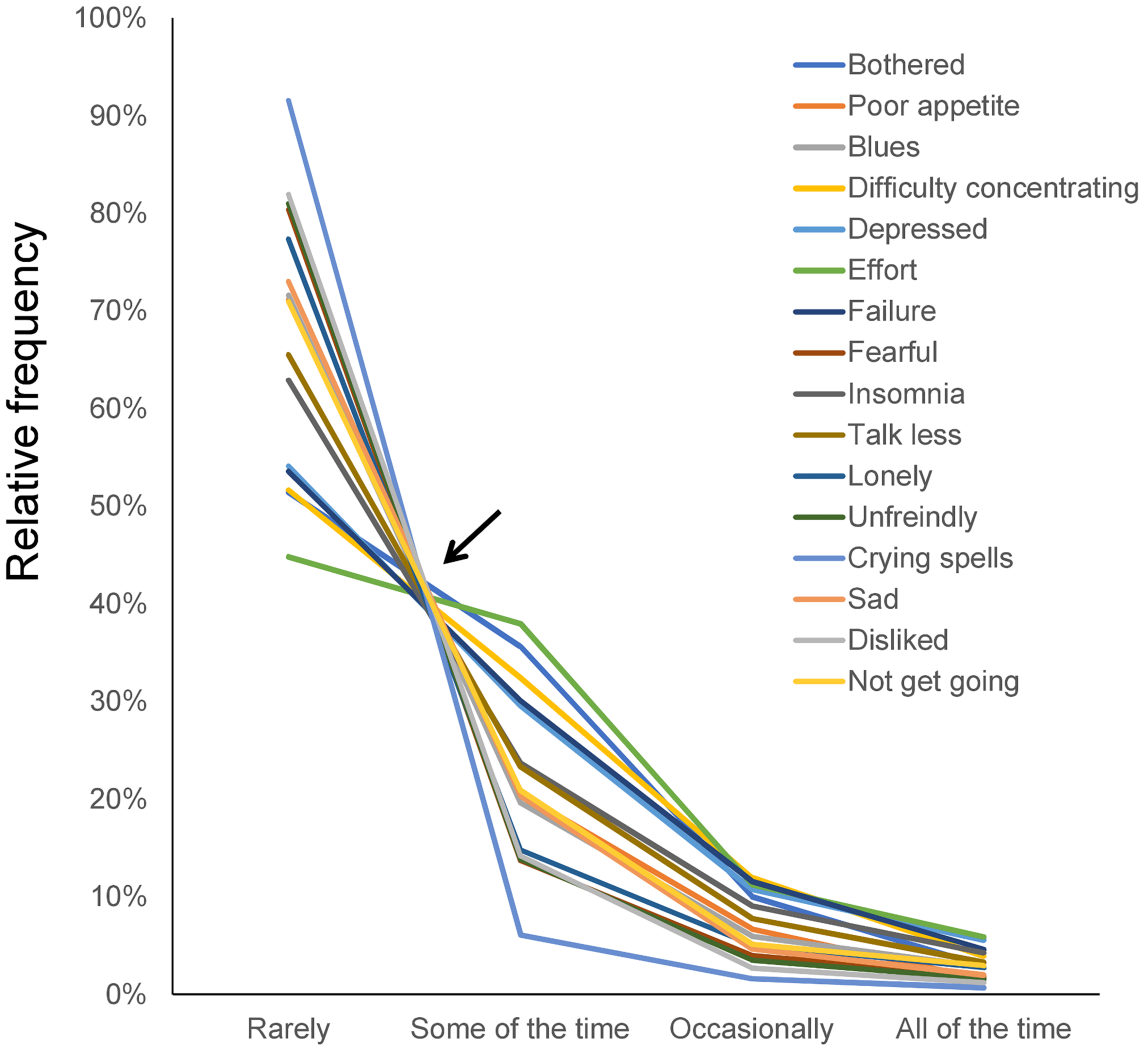
Item responses for the 16 depressive symptom items included in the Japanese survey. Responses to the 16 items exhibited a common mathematical pattern. The lines for the 16 items intersect at a single point (black arrow) between “rarely” and “some of the time,” while the lines between “some of the time” and “all of the time” decrease regularly. Reprinted from [9]. Image credit: PLoS ONE at https://doi.org/10.1371/journal.pone.0165928.g001.

**Fig 2:**
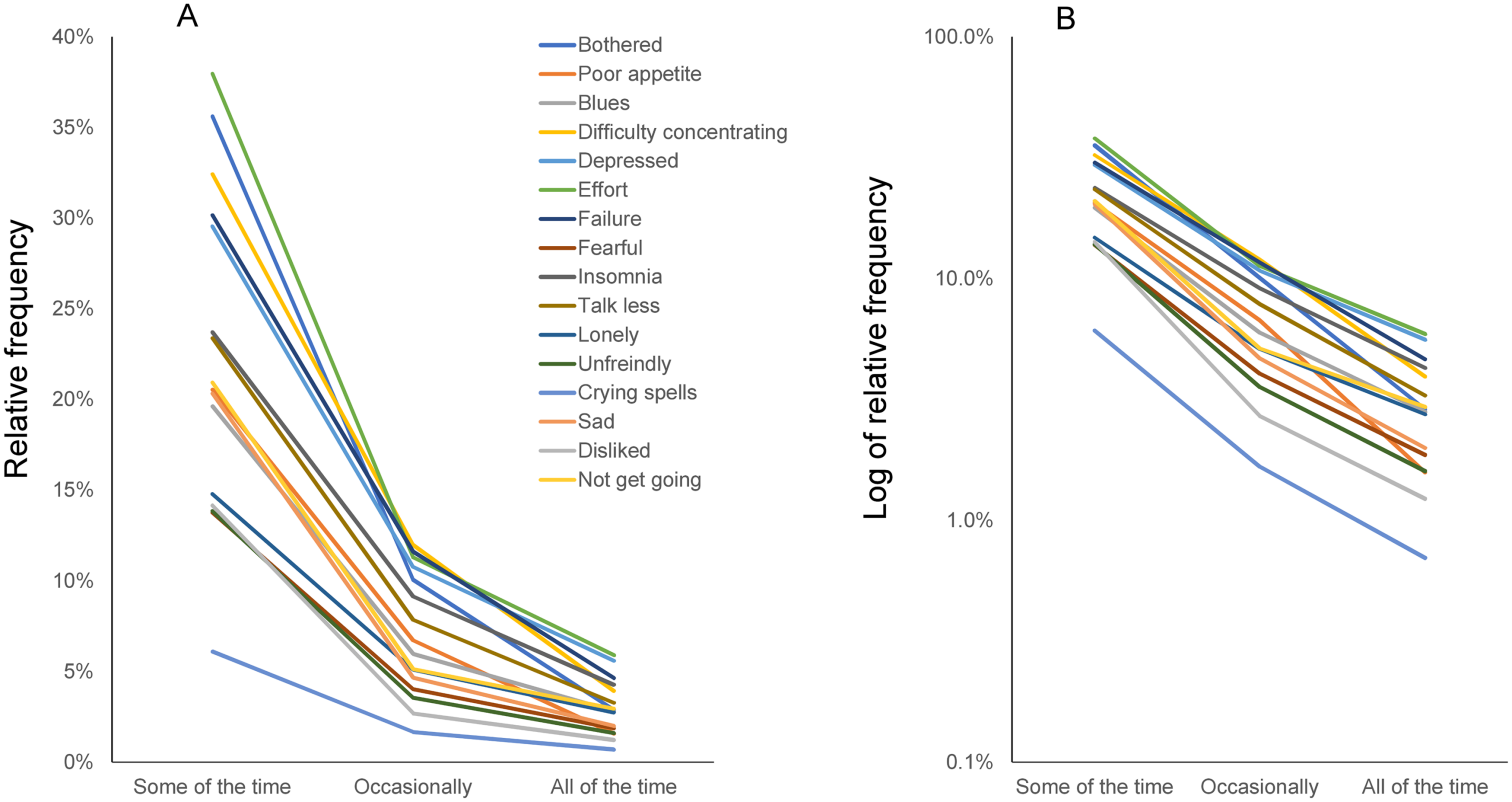
Item responses for the 16 depressive symptom items from “some of the time” to “all of the time” in the Japanese survey. Item responses to the 16 items from “some of the time” to “all of the time” on a normal scale (A) and a log-normal scale (B). (A) The lines for the 16 items converge between “some of the time” and “all of the time.” (B) Item responses follow a parallel pattern between “some of the time” and “all of the time” on a log-normal scale. Reprinted from [9]. Image credit: PLoS ONE at https://doi.org/10.1371/journal.pone.0165928.g001.

In a previous study, we analyzed CES-D data from nearly 32,000 participants enrolled in a Japanese national survey. We found that item responses to the CES-D exhibited a common pattern among the 16 depressive symptom items in this population (Fig 1) [8,9]. The response-curve lines for item responses to these 16 depressive symptom items intersected at a single point between “rarely” and “some of the time” but infrequently intersected between “some of the time” and “all of the time.” The lines for item responses decrease regularly between “some of the time” and “all of the time.” (Fig 2A). When plotted on a log-normal scale, the item response lines for the 16 depressive items followed a parallel pattern between “some of the time” and “all of the time.” (Fig 2B). Unlike the 16 depressive symptom items, the remaining four positive affect items did not exhibit a specific pattern [8].

The characteristic pattern of item responses has been replicated in previous studies using other depression screening scales. Analyses of Patient Health Questionnaire-8 (PHQ-8) data from the Behavioral Risk Factor Surveillance Survey and PHQ-9 data from the National Health and Nutrition Examination Survey (NHANES) in the United States have also revealed this characteristic pattern [10,11]. Moreover, analyses of four subsamples from the Nationally Survey of Midlife Development in the United States (MIDUS) confirmed that item responses to the Kessler Screening Scale for Psychological Distress (K6) followed this same characteristic pattern in a population of US adults [12].

Of note, previous studies have demonstrated that the ratios between two consecutive response options were similar among all depressive symptom items, with the exception of response options at the lower end of the curve [8,10,12]. In addition, these similar ratios between two consecutive response options have been shown to result in the same characteristic pattern of item responses described above: response-curve lines cross at a single point between the option at the lower end and the adjacent option, with a parallel pattern across the remaining options on a log-normal scale. [8,9].

Furthermore, as with response options, total scores on such scales have also been reported to follow a characteristic distribution pattern in the general population. In the analysis of the same surveys of the representative Japanese population sample described above, we found that total CES-D scores followed an exponential pattern, except for at the lower end of the distribution [13]. These findings have been replicated in a sample from the British National Household Psychiatric Morbidity Survey using the Revised Clinical Interview Schedule (CIS-R) [14], the same sample from the NHANES using the PHQ-9 [11], and the same four subsamples from the MIDUS using the K6 [12].

Taken together, these findings suggest that item responses and total score distributions on depression screening scales exhibit common, characteristic patterns in the general population. If it is established that item responses and total scores follow a non-normal distribution, the statistical procedures assuming normal distributions (e.g., parametric statistics) will require reconsideration. However, much of the research on frequency distribution patterns in depression screening scales, especially with regard to item responses, has been limited to data from US- and Japan-based populations. The degree to which these findings can be generalized to other national populations is unclear and therefore warrants examination.

The Irish Longitudinal Study on Ageing (TILDA) is a biennial and longitudinal survey that provides nationally representative estimates of health status variables among the elderly in Ireland [15]. Ireland is a Celtic nation in northwestern Europe and its population is estimated at just over 4.8 million. Ireland has a relatively homogeneous population and its ethnicity, culture, and religion differ from those of the United States and Japan [15]. It is important to confirm whether item responses and total score distributions on depression screening scales exhibit the same characteristic patterns in this population regardless of the differences in ethnicity, culture, and religion.

CES-D is included as part of the TILDA, from which de-identified data are available for researchers worldwide through the Inter-university Consortium for Political and Social Research [16]. TILDA data provide a large sample seize, enabling studies, such as the one conducted here, which require significant statistical power. Generally speaking, the distribution across a large sample size more closely approximates a theoretical distribution due to convergence in distribution [17]. Through analyses of the 2009–2011 TILDA data, we sought to elucidate the characteristics of item responses and total score distributions on the CES-D in an older Irish population, and successfully identified the characteristic patterns in this population [8,13].

## Materials and methods

### Dataset

This study used the data from the 2009–2011 TILDA Wave 1 [16]. The TILDA is a nationally representative, longitudinal study led by Trinity College Dublin in collaboration with other principal academic institutions in Ireland [15]. The purpose of the TILDA is to assess the health, social, and financial circumstances of older Irish adults using a questionnaire which consists of detailed questions on health, social, and financial circumstances. Eligible respondents for the survey consist of individuals aged 50 and over and/or their partners in Ireland. The TILDA sample included a small number of respondents under the age of 50 because partners of eligible respondents (ages 50 and over) were sometimes under the age of 50. Participants were selected to represent the elderly Irish population more broadly on the bases of stratification, multi-stage selection, and representative probability sampling. The household response rate to the TILDA was 62% [15].

The TILDA sample used here consisted of 8,504 respondents (ages 49 and younger, *N* = 329 [male: *n* = 36]; ages 50–59 years, *N* = 3271 [male: *n* = 1461]; ages 60–69 years, *n* = 2589 [male: *n* = 1206]; ages 70–79 years, *n* = 1677 [male: *n* = 804]; ages 80 years or older, *n* = 626 [male: *n* = 268]; ages missing, *n* = 12 [male: *n* = 10]). Sociodemographic characteristics of participants in the 2009–2011 TILDA have been reported in detail elsewhere [15].

### Study cohort

Among the 8,504 respondents from the TILDA sample, individuals who did not answer all CES-D items were excluded from the present analyses. The excluded sample comprised 133 individuals (1.5%), yielding a final sample of 8371 individuals.

### Ethics statement

Ethical approval for the TILDA was obtained from the Trinity College Research Ethics committee in Ireland and participants provided written informed consent. The present paper analyzes de-identified TILDA data, which are available for researchers worldwide. The ethics committee of the Panasonic Health Center does not consider de-identified public data analysis to be a form of human subjects’ research, and as such our project did not require the committee’s approval. The requirement for patient consent to the present study was waived due to the same reason.

### Measures

In the 2009–2011 TILDA, participants’ depressive symptoms were assessed using the CES-D. The CES-D includes 16 depressive symptoms items and four positive affect items. Symptoms the participants experienced during the week prior to assessment were classified on a four-point Likert scale including the following options: 0 = rarely, 1 = some of the time, 2 = occasionally, and 3 = all of the time [7]. Positive affect items were scored in the inverse order (e.g., 0 = all of the time). Previous studies have demonstrated that the distributions of these 16 depressive symptoms items exhibit a characteristic pattern, while the distribution of the four positive affect items do not [8,18]. Given this, we analyzed patterns associated with both item responses and total scores for the 16 depressive symptom items. The total possible item score for the 16 depressive symptoms was 48.

### Data analyses

First, we analyzed the distributions of item responses for all 16 depressive symptom items. If the ratios between two consecutive response options between “some of the time” and “all of the time” were similar among all items, the item response was concluded to exhibit the previously reported, characteristic pattern [9,12]. Thus, the ratios of “occasionally” to “some of the time” and of “all of the time” to “occasionally” were calculated for all 16 items. Next, we graphically analyzed the patterns of item responses. Although this is the source of some debate, we used line charts and not bar charts in the present study [19]. The strength of line charts is their ability to reveal relationships among discrete, x-axis categories. The patterns of item responses for the 16 depressive symptom items were visualized using normal and log-normal scales.

After confirming that the item responses exhibited the same characteristic pattern among the 16 items as had been previously reported in different populations [8,18], the distribution pattern of the total scores for the 16 depressive items was analyzed via graphical analysis and exponential regression modeling. A log-normal scale was employed to enable exponential pattern detection, which is linear along this scale. Exponential regression curves were estimated using the least squares method. All statistical analyses were performed using JMP Version 11 for Windows (SAS Institute, Inc., Cary, NC, USA).

## Results

### Item response analyses

Item response rates demonstrated a similar pattern among the 16 items—the highest response rate being for “rarely” and a decreasing response rate thereafter as item scores increased, with the lowest response rate being for “all of the time” (Table 1). The decreasing ratio of “some of the time” to “occasionally” ranged from 0.24 to 0.47, and the decreasing ratio of “all of the time” to “occasionally” ranged from 0.24 to 0.68. These ratios were not markedly different among the 16 items. The average ratio of “some of the time” to “occasionally” (0.35 ± 0.06) was lower than that of “all of the time” to “occasionally” (0.42 ± 0.15).

**Table 1:** Center for Epidemiologic Studies Depression Scale (CES-D) item responses.

No	Item	Item response (%)				Rate of "2" to "1"	Rate of "3" to "2"
		0	1	2	3		
1	Bothered	80.0%	13.0%	5.3%	1.8%	0.41	0.34
2	Poor appetite	88.3%	7.6%	3.0%	1.1%	0.39	0.36
3	Blues	86.9%	9.1%	3.0%	1.0%	0.33	0.35
5	Difficulty concentrating	73.9%	17.6%	6.8%	1.6%	0.39	0.24
6	Depressed	82.3%	11.9%	4.5%	1.3%	0.37	0.30
7	Effort	79.4%	13.7%	4.4%	2.5%	0.32	0.57
9	Failure	91.2%	5.4%	2.0%	1.4%	0.38	0.68
10	Fearful	85.0%	10.8%	3.1%	1.1%	0.29	0.36
11	Insomnia	59.8%	22.3%	10.6%	7.2%	0.47	0.68
13	Talk less	84.8%	10.4%	3.5%	1.3%	0.33	0.39
14	Lonely	81.5%	11.6%	5.1%	1.9%	0.44	0.37
15	Unfriendly	91.4%	6.1%	1.6%	0.8%	0.27	0.51
17	Crying spells	88.9%	7.6%	2.7%	0.8%	0.35	0.29
18	Sad	74.1%	18.9%	5.6%	1.4%	0.30	0.25
19	People dislike me	93.6%	4.5%	1.1%	0.7%	0.24	0.68
20	Could not get going	80.6%	13.9%	4.0%	1.5%	0.29	0.37
Average		82.6%	11.5%	4.1%	1.7%	0.35 ± 0.06	0.42 ± 0.15

*Note*. Each of the 16 items is scored with one of four options: 0 (*rarely*), 1 (*some of the time*), 2 (*occasionally*), and 3 (*all of the time*). Average rate data are presented as mean plus or minus one standard deviation.

**Fig 3:**
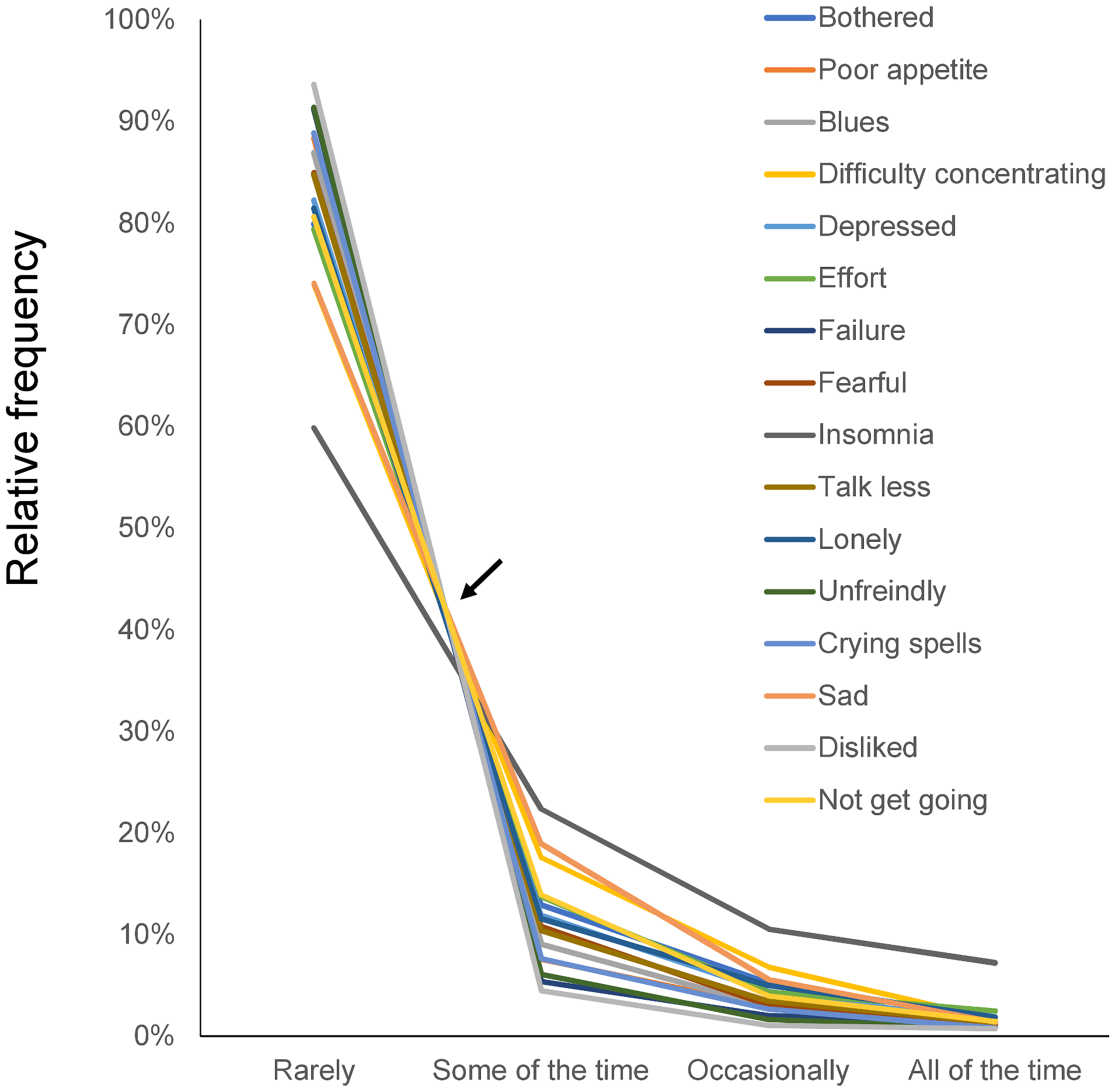
Item responses of the 16 depressive symptom items in the TILDA. Responses to the 16 items exhibited a common mathematical pattern. Lines for the 16 items intersected at a single point (arrow) between “rarely” and “some of the time,” whereas the lines from “some of the time” to “all of the time” decrease regularly. The line for “insomnia” intersected distal to the intersection point.

To demonstrate the pattern of item responses, we plotted all 16 item response rates together on a single graph (Fig 3). The item responses showed a common pattern across the 16 items. Lines for the 16 items intersected around a single point between “rarely” and “some of the time,” after which, they decreased similarly. The line for “insomnia” appeared to intersect distal to the point of convergence.

**Fig 4:**
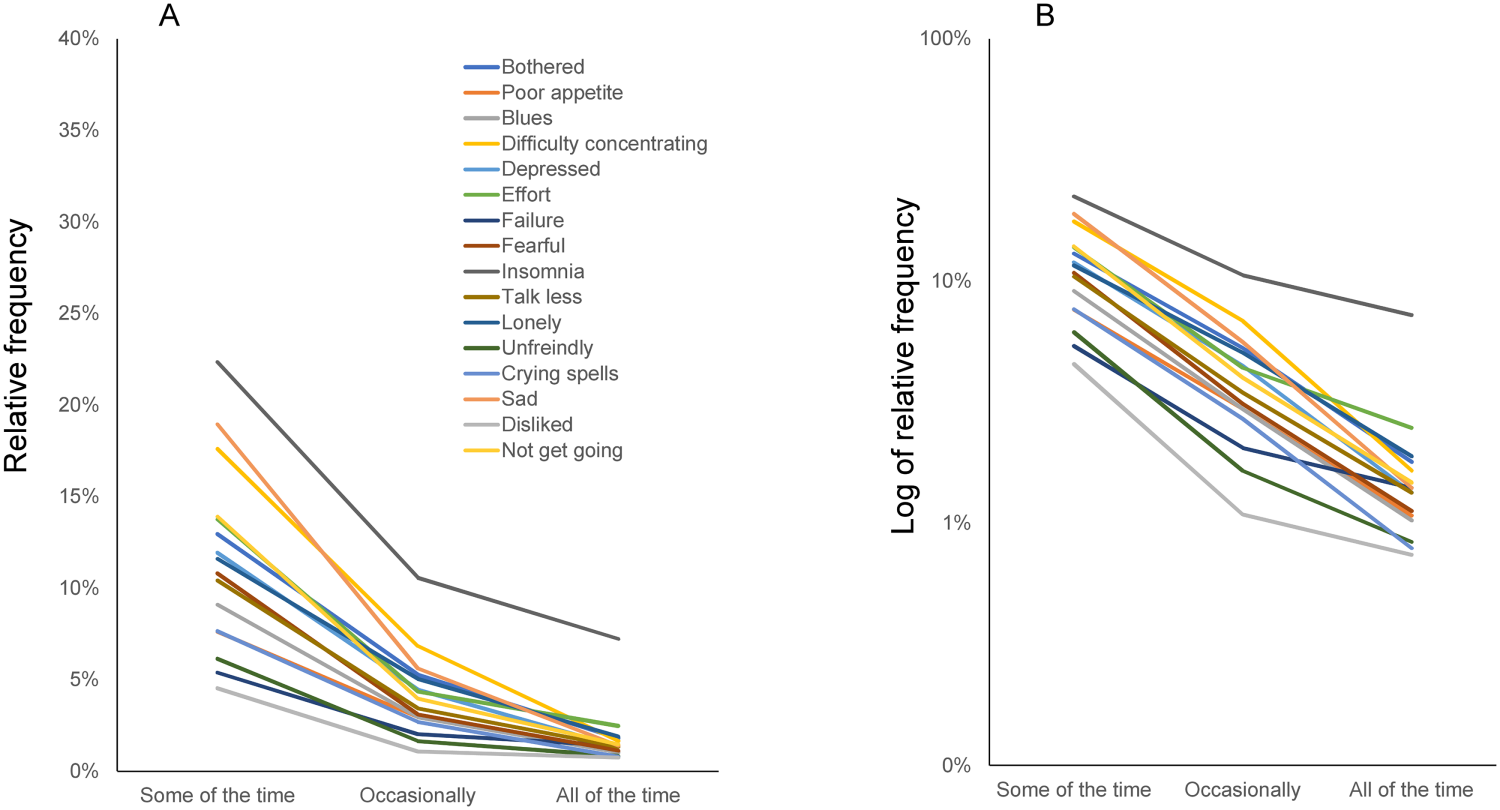
Item responses to the 16 depressive symptom items from “some of the time” to “all of the time.” Responses to the 16 items from “some of the time” to “all of the time” along a normal scale (A) and a log-normal scale (B). (A) Lines for the 16 items converged from “some of the time” to “all of the time.” (B) Most of the item responses for the 16 items follow a parallel pattern between “some of the time” and “all of the time.” The lines for “insomnia,” “failure,” and “dislike” were not in such a parallel pattern as that seen for the other items.

Between “some of the time” and “all of the time,” all lines representing item responses converged as item scores increased (Fig 4A), consistent with previous studies (Fig 2A). One exception was the line for “insomnia,” which did not converge with the others between “occasionally” and “all of the time” (Fig 4A). Using a log-normal scale, we found that item response lines generally decreased parallelly between “some of the time” and “all of the time” (Fig 4B). As reported previously, the degree of parallelism of these 16 lines reflects how decreasing ratios of “occasionally” to “some of the time” and of “all of the time” to “occasionally” are similar among the 16 depressive item scores after logarithmic transformation [8]. Because log-normal scales represent these decreasing ratios after logarithmic transformation, small differences in the ratios between two consecutive options do not have a great effect on the parallelism of the 16 lines [10]. Unlike the lines between “some of the time” and “occasionally,” some of the lines (“insomnia,” “failure,” and “dislike”) between “occasionally” and “all of the time” were further from parallel (Fig 4B).

### Total score analyses

**Fig 5:**
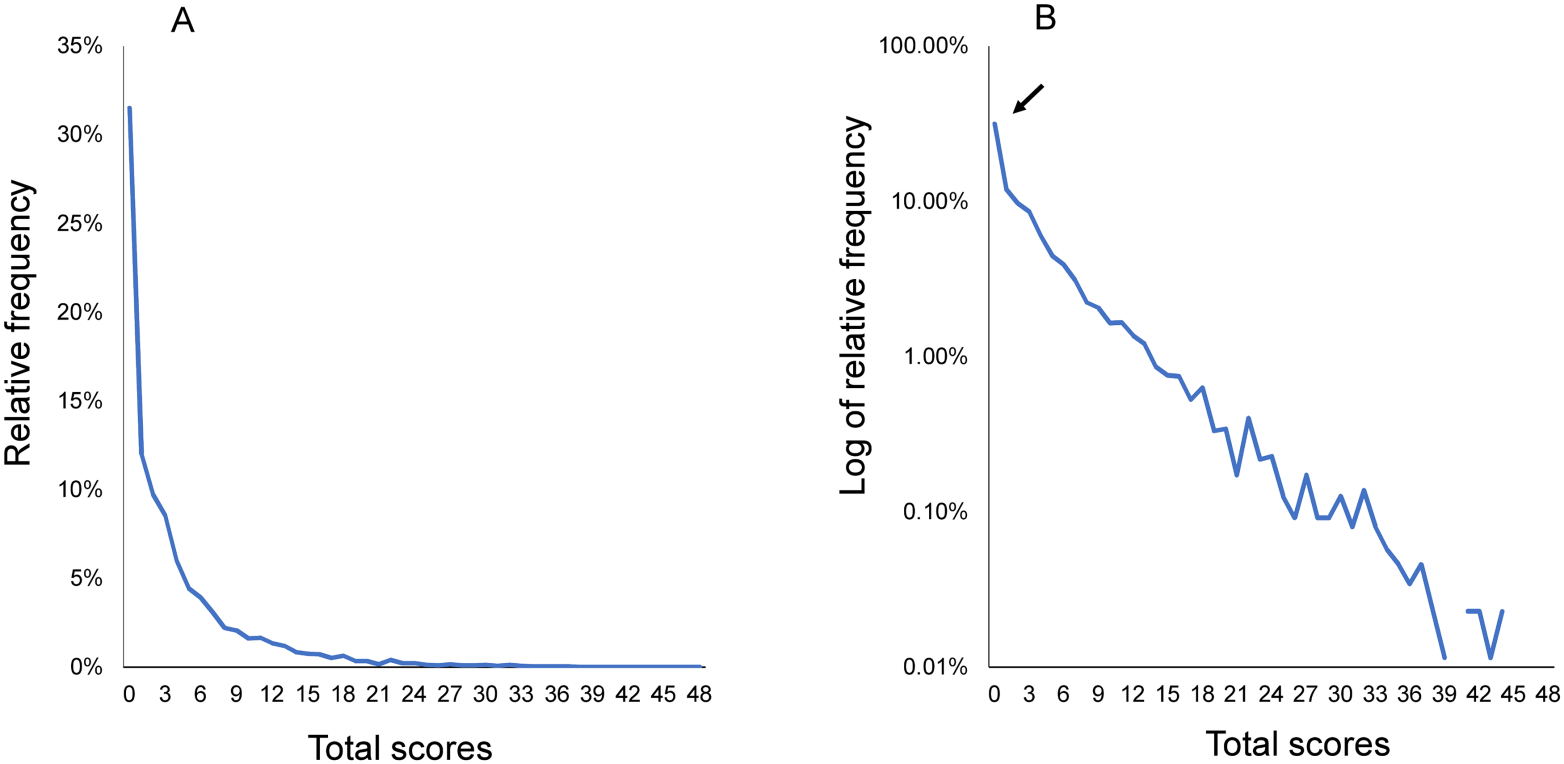
Total score distribution of the 16 items on normal and log-normal scales. Total score distribution of the 16 items along a normal scale (A) and a log-normal scale (B). (A) The total score distribution of the 16 items was right-skewed. (B) The total score distribution for the 16 items followed an exponential pattern (linear pattern), except for at the lower end of the distribution. The distribution at the lower end (arrow) exhibited higher frequencies compared to those expected for an exponential pattern.

The distribution of total scores for the 16 depressive symptom items was right-skewed and the relative frequency of the zero score was 31.5% (Fig 5A). Using a log-normal scale, we found that the distribution was linear, suggesting that the total scores of the 16 items followed an exponential pattern (Fig 5B). The distribution of total scores fluctuated more as total scores increased, reflecting the smaller sample sizes among higher scores. Furthermore, the distribution diverted slightly from an exponential pattern at its lower end (arrow).

Regression curves for an exponential model were calculated for the distribution between the full possible range of 0–48 points (y = 0.125e^-0.16x^, R^2^ = 0.97). The independent variable (x) and dependent variable (y) represented the K6 total score and the relative frequency of participants, respectively. R^2^ was the coefficient of determination. Analysis revealed high coefficients of determination, suggesting a good fit of the distribution of total scores to an exponential distribution.

## Discussion

The aim of the present study was to investigate the patterns of item responses and total score distributions of 16 depressive symptom items from the CES-D using TILDA cohort data. Two primary findings are revealed here: (1) item responses exhibited the same characteristic pattern among the 16 items and (2) the total item scores showed an exponential pattern, except for at the lower end of the distribution. These findings are consistent with those of previous studies conducted in the United States and Japan and support the hypothesis that the item responses and total scores on depression screening scales follow the same characteristic patterns when assessed in a general population [8,13].

The results described here, as well as previous results, show that item responses and total score distributions on depression screening scales follow a non-normal distribution. However, statistical procedures that assume a normal distribution (e.g., parametric statistics and factor analysis) have been widely used to analyze depression screening scale data in the general population. To our knowledge, there has been little evidence that the item responses and total score distributions on depression symptom scales follow a normal distribution in the general population [7]. Our findings suggest that statistical procedures assuming a normal distribution may be inappropriate for depression screening scale data analysis in the general population.

It remains unclear why item responses on depression screening scales in the general population exhibit the characteristic pattern described here and elsewhere. From a mathematics standpoint, if the ratio between two consecutive response options with the exception of response options at the lower end is similar across all items, responses should follow this characteristic pattern [8]. In fact, the ratios between “some of the time” and “occasionally” and between “all of the time” and “occasionally” were similar to some extent among the 16 survey items assessed in the present study. Further studies are necessary to fully clarify the relationship between the responses to these items.

The present results provide further evidence that total depression screening scale scores among the general population approximate an exponential distribution, except for at the lower end of the distribution and irrespective of the scale used [12,14]. The reason for an exponential pattern for total scores is unclear, although an exponential distribution generally results from both individual variability and total stability (i.e., maximum entropy) [20,21]. The results of a recent simulation study conducted by our group agree with the results described here, demonstrating that if a latent depressive symptom trait approximates an exponential distribution, total depression screening scores exhibit an exponential pattern, except for at the lower end of the distribution [22]. In the present study, the total score distribution diverted slightly from an exponential pattern at its lower end (Fig 5B), consistent with the results of previous studies [13,18].

Some noteworthy differences exist between the results ascertained from the TILDA cohort here and from a previous depression-scale study of the general Japanese population. First, the average response rates for “some of the time” (11.5%), “occasionally” (4.1%), and “all of the time” (1.7%) were lower in the TILDA dataset (Fig 4A) compared to those for “some of the time” (22.3%), “occasionally” (7.0%), and “all of the time” (3.0%) in Japanese survey data (Fig 2A) [9]. These findings may reflect prior findings that CES-D scores are generally higher in the general population in Eastern Asia than in Western countries [23]. Next, compared with the previous results from the Japanese survey (Figs 1 and 2B), the line for “insomnia” was slightly removed from the single convergence point for other items. It was also further from parallel between the “occasionally” and “all of the time” responses (Figs 3 and 4B). These differences may be related to a high “all of the time” response rate for “insomnia” in the TILDA dataset (Fig 4A). In general, the incidence of insomnia increases and symptoms worsen with age. While the TILDA cohort predominantly consists of individuals 50 years or older, the Japanese survey consists of individuals of all ages (over 12 years). These results suggest that participant age profiles may account for a higher “all of the time” response rate for “insomnia” in the TILDA dataset.

This analysis has some limitations and strengths that warrant further discussion. Although we investigated whether the item responses and total item scores on the CES-D followed the characteristic patterns observed in previous studies, we did not quantify the fit of the present models of item responses to the TILDA data. As response item patterns were complex, it was difficult to apply a unitary regression analysis. Despite this limitation, our use of graphical analyses using a line graph enabled us to identify a complex pattern of item responses, a significant advantage of this method. This complex pattern would have been overlooked if exact values were presented in table format only. A data table is a good way of displaying exact values but can insufficiently convey underlying patterns in a dataset [24,25].

To the best of our knowledge, this is the first study to demonstrate the characteristic pattern of item responses reported by others from self-reported depression screening scales using European population data. The present study contributes further evidence on the distribution of item responses and total scores on such scales across the general population. Although there is some debate, the fact that intelligence test scores approximate a normal distribution is one of the great discoveries in psychology [26]. Similarly, the specific patterns of item scores and total scores on such scales could contribute to our understanding of how depressive symptoms are distributed across the general population.
